# Combinational Therapy of Mesenchymal Stem Cells and Metformin in Bleomycin-Induced Idiopathic Pulmonary Fibrosis in Rat Model

**DOI:** 10.1007/s12010-025-05289-y

**Published:** 2025-06-21

**Authors:** Nourhan Hassan, Mariam N. Elbyoume, Mariam A. Taha, Hagar S. Mohamed, Omnia M. Elmoghini, Shorouk S. Raouf, Rwan K. Elsayem, Mohrail M. Medhat, Razan M. Rostom, Mohmed Hosney, Emad M. Elzayat

**Affiliations:** 1 Department of Biotechnology, Faculty of Science, Cairo University, Cairo, 12613 Egypt; 2 Biotechnology/Biomolecular Chemistry Program, Faculty of Science, Cairo University, Giza, 12613 Egypt; 3 Zoology Department, Faculty of Science, Cairo University, Giza, 12613 Egypt

**Keywords:** Idiopathic pulmonary fibrosis, Mesenchymal stem cells, Collagen deposition, Metformin, Bleomycin

## Abstract

Idiopathic pulmonary fibrosis (IPF) is a progressive and severe lung disease characterized by the buildup of interstitial fibrosis, where excessive collagen accumulates, leading to airway obstruction. This condition is initiated by the abnormal proliferation of alveolar type II (AT2) cells. Metformin, an established antidiabetic drug, has gained attention for its repurposed use as an anti-fibrotic agent. Meanwhile, adipose-derived mesenchymal stem cells (ADMSCs) exhibit potent anti-inflammatory and regenerative properties, and they have been shown to reduce collagen deposition. In this study, we hypothesize that the combination of metformin and ADMSCs can synergistically alleviate IPF and promote healthy lung tissue regeneration in a rat model. The goal is to evaluate the safety and efficacy of this approach at multiple levels; biochemical, molecular, histopathological, and histochemical. To induce IPF, Wistar albino rats received a single intratracheal dose of bleomycin (5 mg/kg body weight). The therapeutic phase involved treatment with either metformin or ADMSCs or a combination of both. Metformin was administered intraperitoneally (65 mg/kg body weight) every other day, while ADMSCs were delivered intravenously (1 × 10⁶ cells/0.5 ml DMEM/rat) through the tail vein. Our results demonstrated the effectiveness of combinational therapy, especially in mitigating oxidative stress. This was evidenced by the restoration of oxidative stress biomarkers, malondialdehyde (MDA), and catalase (CAT), as well as the regulation of collagenase type IV (MMP9), bovine serum albumin (BSA), and total protein levels in lung tissues. Moreover, the therapy modulated the expression of key inflammatory and fibrotic genes, including the pro-fibrotic marker TGF-β1, proinflammatory cytokine IL-6, and anti-inflammatory cytokine IL-10. Histopathological and histochemical analyses further supported the therapeutic benefits, showing significant recovery from bleomycin-induced fibrosis in rats treated with either the single or combined therapy. The findings suggest that this combinational approach could be a promising strategy for IPF treatment by simultaneously reducing inflammation, oxidative stress, and fibrosis while promoting tissue regeneration.

**Graphical Abstract:**

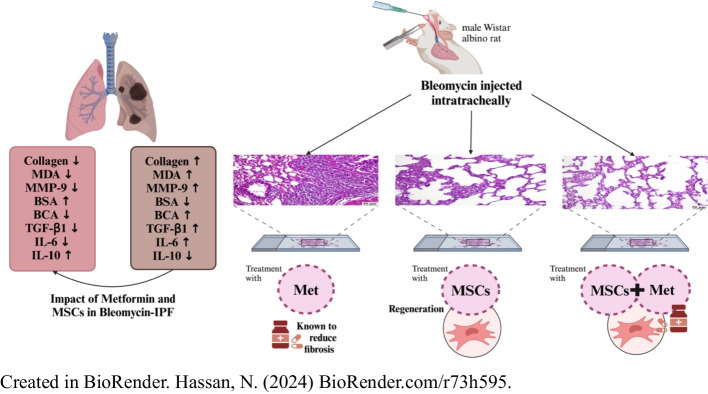

## Introduction

Idiopathic pulmonary fibrosis (IPF) is a chronic and progressive lung disease characterized by the gradual development of scar tissue (fibrosis) within the lungs, leading to significant breathing difficulties and reduced lung function over timestamps including persistent shortness of breath, chronic coughing, crackling sounds upon inhalation, and finger clubbing [1]. Although the disease progression can vary among patients, it typically leads to a marked decline in respiratory function, severely affecting the quality of life and, in many cases, resulting in fatal outcomes [2–4]. Globally, IPF affects approximately 3 million individuals, with the incidence rising notably with age, especially in people over 50 [4, 5]. The diagnosis is difficult and often delayed as there are no definitive clinical tests and due to its symptomatic overlap with other respiratory diseases such as asthma, chronic obstructive pulmonary disease (COPD), and pneumonia. This similarity can lead to initial misdiagnosis and postponement of appropriate treatment [2, 3]. This diagnostic challenge underscores the need for increased awareness and advancements in diagnostic tools, which could enable earlier and more accurate disease identification, ultimately improving patient outcomes.

The precise mechanisms driving fibrosis in IPF are not yet fully understood. However, current theories suggest that the disease originates from repeated, subclinical injuries to a genetically susceptible alveolar epithelium, which fails to adequately regenerate and repair itself [6, 7]. This dysfunction triggers a cascade of events within the alveoli, where activated alveolar type II (AT2) cells release various cytokines and growth factors [8]. These signaling molecules promote the recruitment, proliferation, and differentiation of lung fibroblasts into myofibroblasts. The persistent activation of myofibroblasts results in excessive collagen deposition, progressive scarring of lung tissue, and the eventual loss of lung function due to impaired tissue elasticity and gas exchange [8]. Traditional treatments for IPF have primarily focused on the use of oral anti-fibrotic drugs, namely pirfenidone and nintedanib [7]. These medications are designed to alleviate symptoms and slow the progression of the disease. Pirfenidone acts as a broad inhibitor of several pro-fibrotic cytokines, including transforming growth factor-beta 1 (TGF-β1) and platelet-derived growth factor receptor (PDGFR) [7, 9, 10]. By inhibiting these key pathways, pirfenidone helps to limit the activation of fibroblasts and the production of extracellular matrix components such as collagen, ultimately slowing down the fibrotic process [10]. Nintedanib, on the other hand, is an intracellular tyrosine kinase inhibitor that targets multiple growth factor receptors involved in fibrosis, such as PDGFR, fibroblast growth factor receptor (FGFR), and vascular endothelial growth factor receptor (VEGFR), thereby reducing fibroblast activation and migration [11, 12]. Although these medications cannot reverse existing fibrosis, they represent crucial advancements in the management of IPF by stabilizing lung function and potentially extending survival.

Stem cell-based therapies have developed into a promising therapeutic field [13, 14]. Among these, mesenchymal stem cells (MSCs) stand out due to their unique and versatile properties. MSCs are characterized by their capacity for self-renewal and their potent regenerative capabilities, which include differentiation into various cell types, such as epithelial and fibroblast-like cells [15–17]. Additionally, MSCs possess significant immunomodulatory properties, enabling them to regulate immune responses, home to sites of injury, and promote tissue repair in damaged organs [18]. One of the key therapeutic advantages of MSCs in the context of fibrotic diseases is their ability to interfere with the fibrotic signaling pathways, specifically the TGF-β1/Smad axis [19, 20]. By inhibiting this pathway, MSCs suppress TGF-β1-induced fibroblast activation, which is a central mechanism driving the excessive collagen deposition and scarring in IPF [21]. This suppression not only reduces fibroblast proliferation but also mitigates collagen deposition, thereby helping to alleviate fibrosis and support the regeneration of healthy tissue [22]. These multifunctional properties of MSCs make them a highly promising candidate for the development of advanced therapies aimed at halting or reversing the progression of fibrosis in IPF and other fibrotic conditions.

On the other hand, metformin, one of the most widely prescribed medications for type 2 diabetes mellitus (T2DM), has been in use for nearly six decades, benefiting over 200 million patients globally [23]. It is renowned not only for its efficacy in controlling blood glucose levels but also for its excellent safety profile and relatively low risk of side effects, making it a staple in diabetes management [24–26]. Recently, the therapeutic potential of metformin has extended beyond diabetes, with increasing interest in its repurposing for the treatment of lung diseases [27] Metformin has shown promising antifibrotic effects by modulating several key metabolic and cellular pathways [23, 28]. Notably, it inhibits the profibrotic action of TGF-β1, suppresses collagen synthesis, and stimulates the lipogenic differentiation of lung fibroblasts derived from IPF patients [29]. This modulation is largely mediated through the activation of AMP-activated protein kinase (AMPK), a critical cellular energy sensor that influences various metabolic processes [29]. Additionally, metformin has been shown to accelerate recovery from bleomycin-induced IPF, further underscoring its potential as an antifibrotic agent. By activating AMPK, metformin downregulates alpha-smooth muscle actin (ACTA2) and collagen production, two critical factors involved in the progression of lung fibrosis [30]. These findings highlight metformin’s multifaceted role of metformin in IPF treatment, offering a promising avenue for therapeutic intervention by targeting both metabolic and fibrotic pathways.

The present study aims to evaluate the therapeutic potential and feasibility of combining MSCs with metformin for the treatment of bleomycin-induced IPF in rat models. This approach will be assessed comprehensively at the biochemical, histopathological, histochemical, and molecular levels to provide a multidimensional understanding of its effectiveness. Ultimately, this research aims to lay the groundwork for innovative, safe, and effective treatment strategies for IPF, with the potential for clinical translation.

## Materials and Methods

### Drugs and Chemicals

Bleomycin vials (BLEOCIP 15, Cipla Pharmaceutical Company, India) are used to induce pulmonary fibrosis in rat models. Metformin (Cidophage, CID Pharma Company, Egypt) was used to investigate its antifibrotic potential in the study. Phosphate-buffered saline (PBS) was supplied by Lonza, Switzerland. All chemicals and reagents used were of analytical grade to ensure precision and reproducibility in the experimental outcomes.

### Experimental Animals

Thirty-five male Wistar albino rats, weighing between 180 and 220 g, were obtained from the animal facility at the National Research Center, Giza, Egypt. Prior to the start of the experiment, the animals were adapted for one week in a temperature-controlled environment (25 °C) with a standard 12-h light/dark cycle to ensure optimal adaptation. They were provided with a standard pelleted diet containing 23% protein. All experimental protocols and procedures were reviewed and approved by the Cairo University Institutional Animal Care and Use Committee (CUIACUC, Egypt, Approval No. CU/I/F/10/24) and conducted in accordance with ARRIVE guidelines and the National Research Council’s Guide for the Care and Use of Laboratory Animals [31, 32].

### Experimental Design

**Fig. 1:**
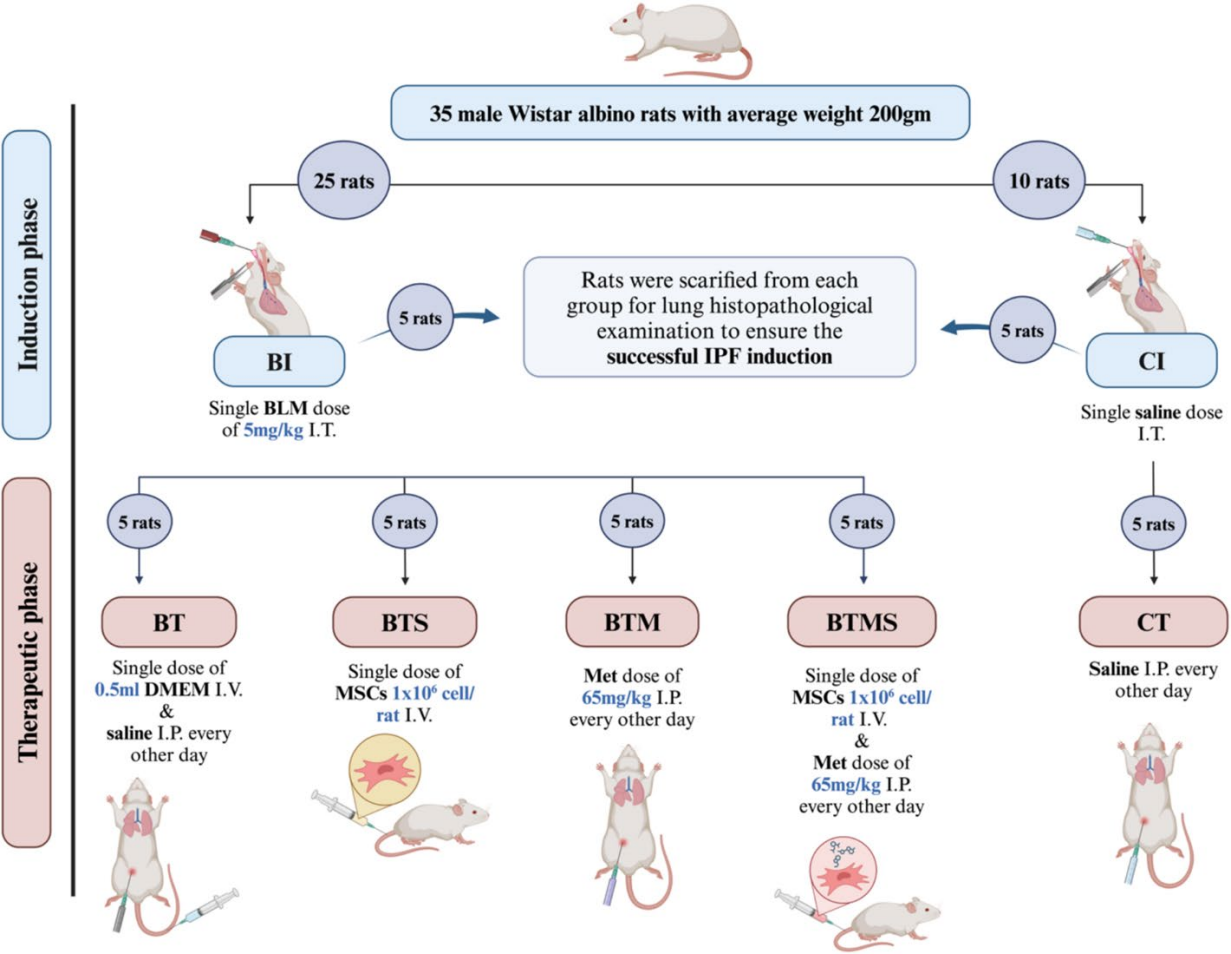
Experimental design for induction and therapeutic phases. In the induction (I) phase, the negative control group is CI, and the bleomycin-induced group is BI. In the therapeutic (T) phase, the negative control group: CT, the bleomycin-induced untreated group: BT, the BT group treated with MSCs: BTS, the BT group treated with metformin: BTM, and the BT group treated with a combination of metformin and MSCs: BTMS. Intratracheally (I.T.)., intraperitoneally (I.P.), and intravenously (I.V.). Created in BioRender. Hassan, N. (2024) https://BioRender.com/l86f060

The experiment was structured into two main phases: an induction phase lasting 51 days and a therapeutic phase of 42 days (Fig. 1). During the induction (I) phase, 35 rats were randomly assigned to two groups. The first group (*n* = 10) served as the negative control (CI) group and received a single dose of saline (2.5 ml/kg) intratracheally (I.T.). The second group (*n* = 25), designated as the bleomycin-induced (BI) group, was administered a single dose of bleomycin (5 mg/kg body weight) intratracheally to induce idiopathic pulmonary fibrosis (IPF) [33]. This dose was selected based on established protocols demonstrating consistent fibrosis development by day 51 without excessive mortality (< 15%) [33, 34]. At the end of the induction phase, five rats from both the control and bleomycin-induced groups were sacrificed for histopathological analysis to confirm the successful induction of IPF. In the therapeutic (T) phase, the remaining five rats in the negative control group (CT) continued the experiment without any treatment, receiving 0.5 ml of normal saline intraperitoneally (I.P.) every other day. Meanwhile, the 20 rats from the bleomycin-induced (BT) group were randomly divided into four subgroups (*n* = 5 each) for treatment: (1) The BT untreated group (received 0.5 ml of DMEM (vehicle for MSCs) once intravenously (I.V.) via the tail vein and 0.5 ml of normal saline (vehicle for metformin) intraperitoneally every other day [29]. (2) The BT group treated with metformin (BTM) (65 mg/kg) intraperitoneally every other day [29, 30]. This dose was optimized from previous antifibrotic studies [29] and showed no adverse effects on weight (maintained > 95% baseline) or blood glucose (90–110 mg/dL) in our pilot experiment. (3) The BT group treated with MSCs (BTS) received a single dose of fluorescently labeled adipose-derived MSCs (1 × 10⁶ cells/0.5 ml/rat I.V. via the tail vein) [30, 35]. (4) The BT group treated with a combination of metformin and MSCs (BTMS) received both metformin (65 mg/kg i.p. every other day) and a single dose of MSCs (1 × 10⁶ cells/0.5 ml/rat I.V.). The staggered administration (MSCs on day 1, metformin starting on day 3) was designed to allow initial MSC homing before pharmacologic modulation [36].

### Isolation, Purification, and Characterization of ADMSCs

ADMSCs were generously provided by Prof. Dr. Laila Rasheed in the Biochemistry Department, Faculty of Medicine, Cairo University from MSC bank. These cells were isolated from the adipose tissues of the pelvic region of rats and cultured in low-glucose DMEM (Lonza, Switzerland) supplemented with appropriate growth factors. Characterization of the ADMSCs was performed using flow cytometry, revealing a positive expression of CD90 and CD29, which are key markers of mesenchymal stem cells. The cells were negative for CD45, confirming their mesenchymal origin and the absence of hematopoietic lineage contamination [37, 38]. The characterization was performed in the Biochemistry Department, Faculty of Medicine, Cairo University and confirmed as previously documented in Abdel Aziz et. al. (2014) [39].

### Blood Collection

Blood samples were collected from the retro-orbital plexus of the rats using heparinized capillary tubes. For plasma separation, blood was collected in tubes pre-coated with EDTA as an anticoagulant [40]. The samples were immediately placed on ice and gently mixed to prevent clotting. The plasma was then separated by centrifugation, and the supernatant (plasma) was then carefully transferred into new tubes. For serum collection, blood samples were drawn into tubes without any anticoagulant and allowed to clot at room temperature for 30 min. After clot formation, the samples were centrifuged, and the resulting serum was carefully transferred to new tubes. Blood serum albumin (BSA) levels were measured spectrophotometrically. All plasma and serum samples were stored at − 80 °C for later further analysis.

### Lung Excision

Following the sacrifice of the rats, both lungs were carefully excised for further analysis. The right lung was divided to be homogenized in PBS and RL buffer at a ratio of 10-mg tissue per 0.6 ml (W/V) and utilized for biochemical and molecular assessments, respectively. Specifically, bicinchoninic acid (BCA) levels were measured using a spectrophotometer to evaluate total protein concentration. The expression of key genes involved in fibrosis and inflammation, including TGF-β1, matrix metalloproteinase 9 (MMP-9), malondialdehyde (MDA), interleukin 6 (IL-6), and interleukin 10 (IL-10), was analyzed using real-time quantitative polymerase chain reaction (RT-qPCR) to assess the therapeutic effects at the molecular level. The left lung was preserved in 10% formal saline for histochemical, histopathological analysis, and the homing of the fluorescently labeled MSCs to the lung tissue.

### Histopathological and Histochemical Examination for Evaluating Lung Tissue

Rats from each experimental group were euthanized, and their left lungs were collected for histopathological analysis. The lung samples were fixed in 10% formal saline, followed by washing, dehydration, clearing, and embedding in paraffin. According to the method described previously [41], 5-µm-thick tissue sections were prepared from the paraffin-embedded blocks. These sections were then stained with hematoxylin and eosin (H&E) to visualize general tissue architecture and identify pathological changes [42]. The stained lung sections were examined under an Olympus BX50 microscope.

Unstained lung tissue slides from the previously prepared sections were also subjected to histochemical staining using Masson’s trichrome (MTC) stain, which is specifically used to detect collagen deposition in tissues. The MTC staining allowed for the visualization and quantification of collagen fibers, an essential marker of fibrosis [43].

### Homing of ADMSCs in Lung Tissues

Two days after the intravenous injection of ADMSCs, lung tissue slides from the experimental groups were examined to track the homing of fluorescently labeled MSCs. Unstained lung tissue slides, as prepared from previously prepared sections, were inspected using a fluorescence microscope (Olympus BX50 microscope). The presence and distribution of MSCs in the lung tissue were visualized.

### Quantification of Lipid Peroxidation, Antioxidant Enzyme Activity, and Protein Levels in Lung Samples

Malondialdehyde (MDA), a key marker of lipid peroxidation and oxidative stress [44], was quantified using a colorimetric ready-made kit (Bio Diagnostic, Egypt) and measured at 534 nm using a spectrophotometer. The activity of superoxide dismutase (SOD), an essential antioxidant enzyme that defends cells against oxidative damage [45], was measured using a SOD ready-made kit (Bio Diagnostic, Egypt) at 560 nm. SOD activity serves as an indicator of the antioxidant defense capacity in lung tissues. Catalase (CAT) activity, another critical component of the antioxidant defense system [46], was measured at 510 nm to evaluate the activity of the enzyme in the lung tissues. Blood serum albumin (BSA) levels were determined using a BSA kit (Bio Diagnostic, Egypt) and measured at 630 nm. BSA levels are a key indicator of protein content in the blood, reflecting overall health and liver function [47].

Matrix metalloproteinase-9 (MMP-9), an enzyme associated with extracellular matrix remodeling and fibrosis [48], was measured in plasma using an enzyme-linked immunosorbent assay (ELISA) (Elabscience, Cat. no. E-EL-R3021, USA). Bicinchoninic acid (BCA) assay was used to measure total protein levels in lung tissue homogenates [49]. The assay was conducted using an ELISA kit (Elabscience Cat. no. E-BC-K318-M, USA) and measured at 562 nm.

### RNA Extraction and qRT-PCR Analysis of Target Genes in Lung Tissue

Total RNA was extracted from lung tissues using the total RNA purification kit (NORGEN, Cat. no. SKU 17200, Canada), following the manufacturer’s protocol. The isolated RNA was then reverse-transcribed into complementary DNA (cDNA) using the EasyScript® First-Strand cDNA Synthesis SuperMix (TransGen Biotech Co, Cat. no. AE301-02, China). GAPDH was employed as a housekeeping gene, ensuring normalization across samples [50]. Target genes included TGF-β1, IL-6, and IL-10, with their forward and reverse primers commercially synthesized by Macrogen (Seoul, South Korea) (Table 1). The reactions were conducted using the HERAPLUS SYBR® Green qPCR Kit (Willowfort, Cat. no. WF10308001, UK) in triplicates. qRT-PCR was performed on an Applied Biosystems StepOnePlus (Thermo Fisher Scientific, Waltham, MA, USA) machine. Relative expression levels of the genes were calculated using the 2 − ΔΔCt method [51].

**Table 1:** The sequences of primers used in qPCR reactions

Gene	Forward primer	Reverse primer
**GAPDH**	5′-AGTGCCAGCCTCGTCTCATA-3′	5′-GATGGTGATGGGTTTCCCGT-3′
**TGF-β1**	5′-ATCCTGAGAGGGCGAGGAAT-3′	5′-GCTGTTAACCGACTTGGGAAC-3′
**IL-6**	5′-GCCCTTCAGGAACAGCTATGA-3′	5′-TGTCAACAACATCAGTCCCAAGA-3′
**IL-10**	5′-GCAGGACTTTAAGGGTTACTTGG-3′	5′-CCTTTGTCTTGGAGCTTATTAAA-3′

### Statistical Analysis

All results were tabulated and expressed as mean ± S.E.M. Statistical analysis was performed using one-way ANOVA followed by Duncan’s post hoc test. Data were analyzed using the statistical package for the social sciences, version 20 (SPSS Inc., Chicago, IL, USA). A *P* value < 0.05 was considered to be statistically significant, and all possible comparisons were made among the study groups.

## Results

### Histopathological Evaluation of Lung Tissue Reveals the Therapeutic Impact of Metformin and MSCs on Bleomycin-IPF

**Fig. 2:**
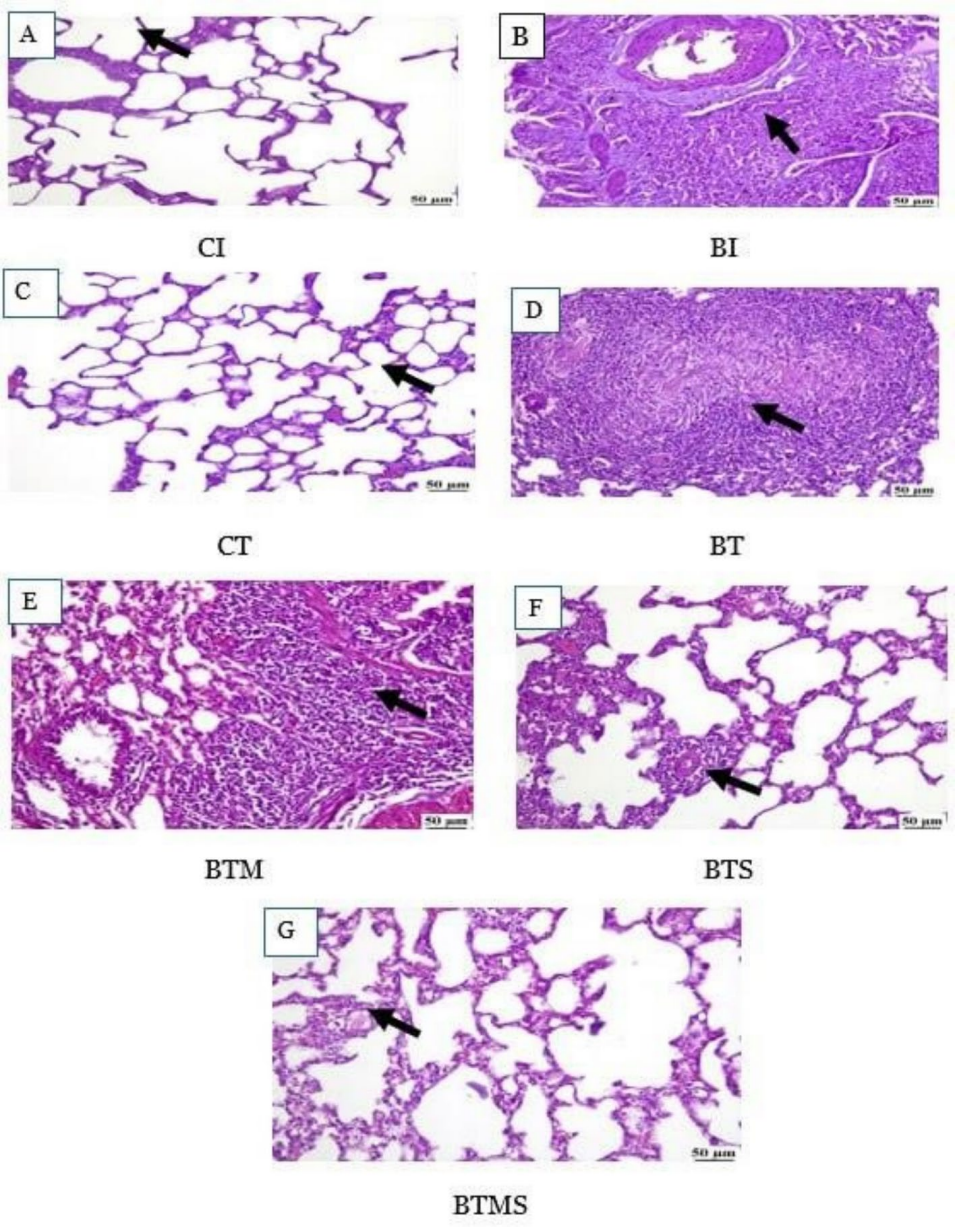
Effect of metformin and/or MSCs on histopathological changes in the lungs of bleomycin-induced rats compared to the control group during the induction and therapeutic phases. In the induction (I) phase, the negative control group: CI, and the bleomycin-induced group: BI. In the therapeutic (T), the negative control group: CT, the bleomycin-induced group: BT, the BT group treated with metformin: BTM, the BT group treated with MSCs: BTS, and the BT group treated with a combination of metformin and MSCs: BTMS. **A** CI group showing normal alveolar structure. **B** BI group exhibiting severe peribronchial and perivascular inflammatory cell infiltration. **C** Lung tissue from the CT group showing normal alveolar structure. **D** BT group displaying severe interstitial pneumonia with fibroplasia. **E** BTM group demonstrating moderate interstitial pneumonia. **F** BTS group showing mild perivascular inflammatory cell infiltration. **G** BTMS group displaying normal lung parenchyma

Histopathological evaluation of lung tissue sections stained with H&E provides critical insights into the structural alterations caused by bleomycin-IPF and the therapeutic effects of metformin and/or MSCs (Fig. 2). In the control groups (CI and CT) (panels A and C), lung tissue maintained a normal histological architecture during both the induction and therapeutic phases. The bronchioles and alveoli exhibited intact structures with no signs of inflammation or fibrosis. In contrast, at the end of the induction phase, the BI group (panel B) displayed marked histopathological changes characterized by intense peri-bronchiolar mononuclear inflammatory cell infiltration and extensive fibroplasia. This damage reflects the successful induction of IPF, marked by fibrosis and inflammation around the bronchioles and alveoli. During the therapeutic phase, the untreated bleomycin group (panel D) exhibited severe multifocal interstitial pneumonia, which included large aggregates of chronic inflammatory cells and pronounced fibroplasia, indicating persistent tissue damage and disease progression.

Treatment with metformin alone (BTM, panel E) resulted in moderate improvement, with the lung sections showing mild interstitial pneumonia and a reduction in inflammatory cell infiltration compared to the untreated BT group. Notably, the BTS group (panel F), which received MSC therapy alone, demonstrated a remarkable reduction in fibroplasia and a further decrease in inflammatory cell infiltration compared to the BTM group.

The combined treatment of MSCs and metformin (BTMS, panel G) showed the most significant improvement, with lung sections displaying nearly normal histological architecture. This improvement underscores the synergistic effect of combining MSCs and metformin, which appears to enhance lung tissue repair and mitigate fibrosis more effectively than either treatment alone.

### Histochemical Analysis Reveals Superior Reduction of Pulmonary Fibrosis with Combined Metformin and MSC Therapy

**Fig. 3:**
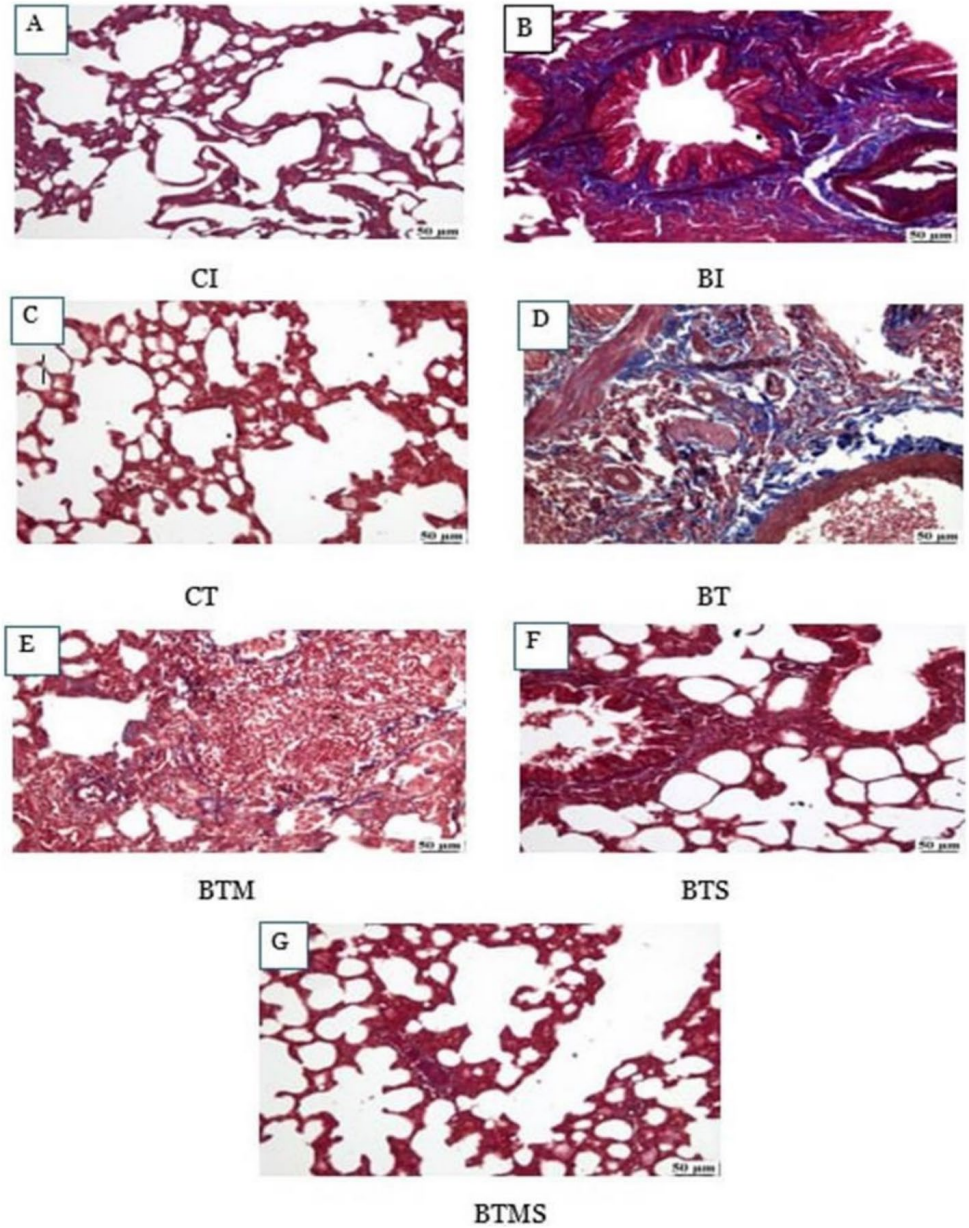
The effect of metformin and/or MSCs on collagen deposition in bleomycin-induced pulmonary fibrosis compared to control groups during the induction and therapeutic phases. In the induction (I) phase, the negative control group: CI, and the bleomycin-induced group: BI. In the therapeutic (T), the negative control group: CT, the bleomycin-induced group: BT, the BT group treated with metformin: BTM, the BT group treated with MSCs: BTS, and the BT group treated with a combination of metformin and MSCs: BTMS. **A** CI group showing normal lung tissue. B BI group showing extensive perivascular and peribronchial fibrosis. **C** CT group showing normal lung tissue. **D** BT group showing interstitial fibrosis. **E** BTM group showing reduced interstitial fibrosis. **F** BTS group showing reduced peribronchial fibrosis. **G** BTMS group showing nearly normal lung tissue

As illustrated in Fig. 3, the histochemical analysis of Masson’s trichrome (MTC)-stained lung sections revealed normal lung parenchyma in both the CI and CT groups, reflecting healthy tissue architecture (panels A and C, respectively). In contrast, the BI and BT groups exhibited marked collagen deposition with pronounced fibrous tissue accumulation, indicative of extensive pulmonary fibrosis (panels B and D, respectively). No significant difference was observed in the area percentage of fibrosis between the BTM and BTS groups, with both treatments showing a moderate reduction in collagen deposition compared to the BT group (panels E and F). Notably, the BTMS group demonstrated the most significant reduction in collagen deposition, approaching normal lung tissue appearance, indicating the superior efficacy of the combined metformin and MSC therapy in mitigating fibrosis (panel G).

### Effects of Metformin and MSCs on Collagen Levels in Bleomycin-IPF

**Fig. 4:**
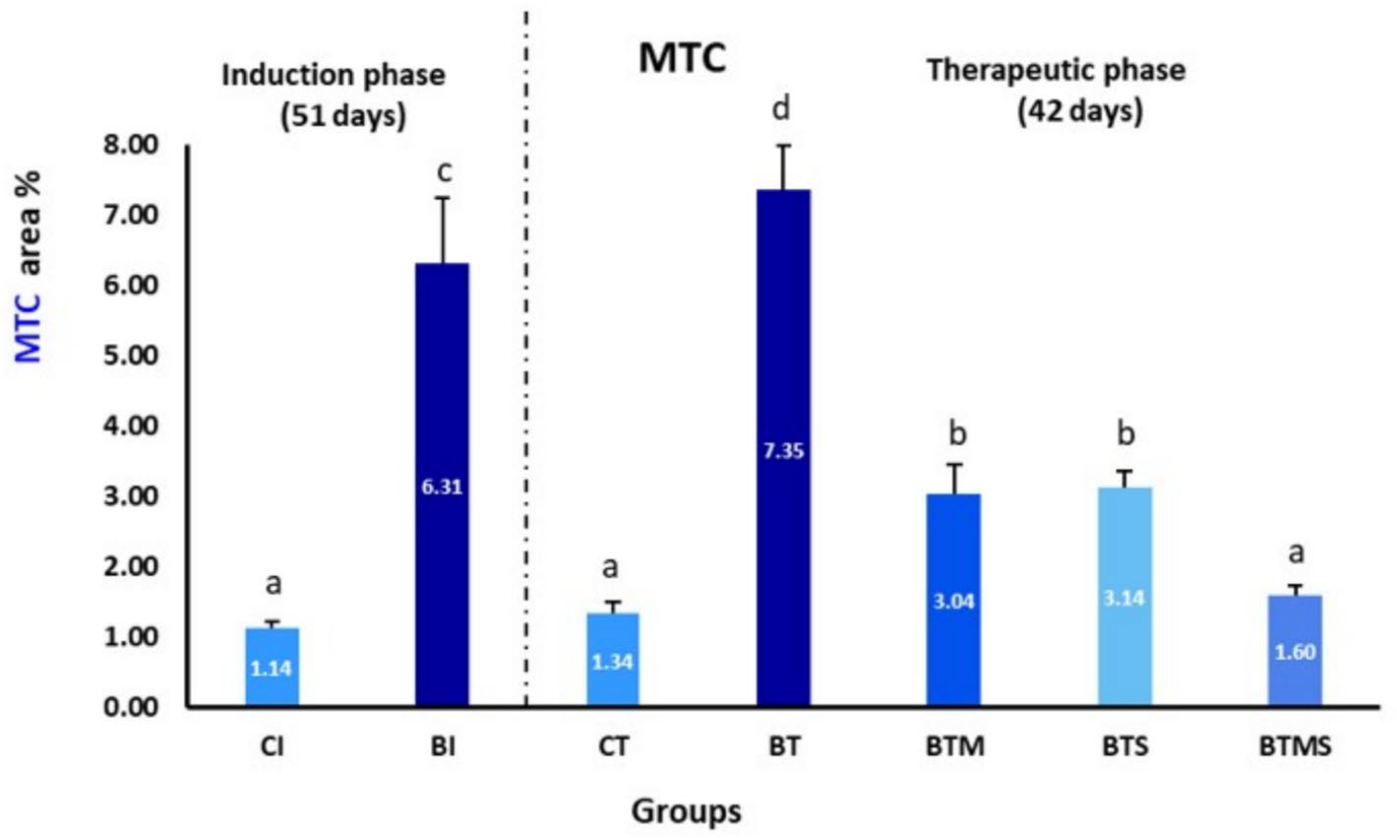
Single and combined effects of metformin and MSCs on collagen levels in lung tissue after bleomycin-induced IPF. In the induction (I) phase, the negative control group: CI, and the bleomycin-induced group: BI. In the therapeutic (T), the negative control group: CT, the bleomycin-induced group: BT, the BT group treated with metformin: BTM, the BT group treated with MSCs: BTS, and the BT group treated with a combination of metformin and MSCs: BTMS. Statistical significance is indicated by different letters where groups with different letters are significantly different at *P* ≤ 0.05, while groups sharing the same letters are not significantly different at *P* > 0.05

Both the BI and BT groups exhibited a significantly higher percentage of collagen deposition compared to their respective control groups (CI and CT) throughout all experimental phases. There was no significant difference in collagen deposition between the BI and BT groups during both the induction and therapeutic phases. In contrast, the BTM and BTS groups demonstrated a significant reduction in collagen deposition. The BTMS group showed marked effectiveness in reducing collagen levels, suggesting a superior therapeutic benefit from the combined treatment approach (Fig. 4).

### In VivoValidation of ADMSCs Homing to Fibrotic Lung Tissue

**Fig. 5:**
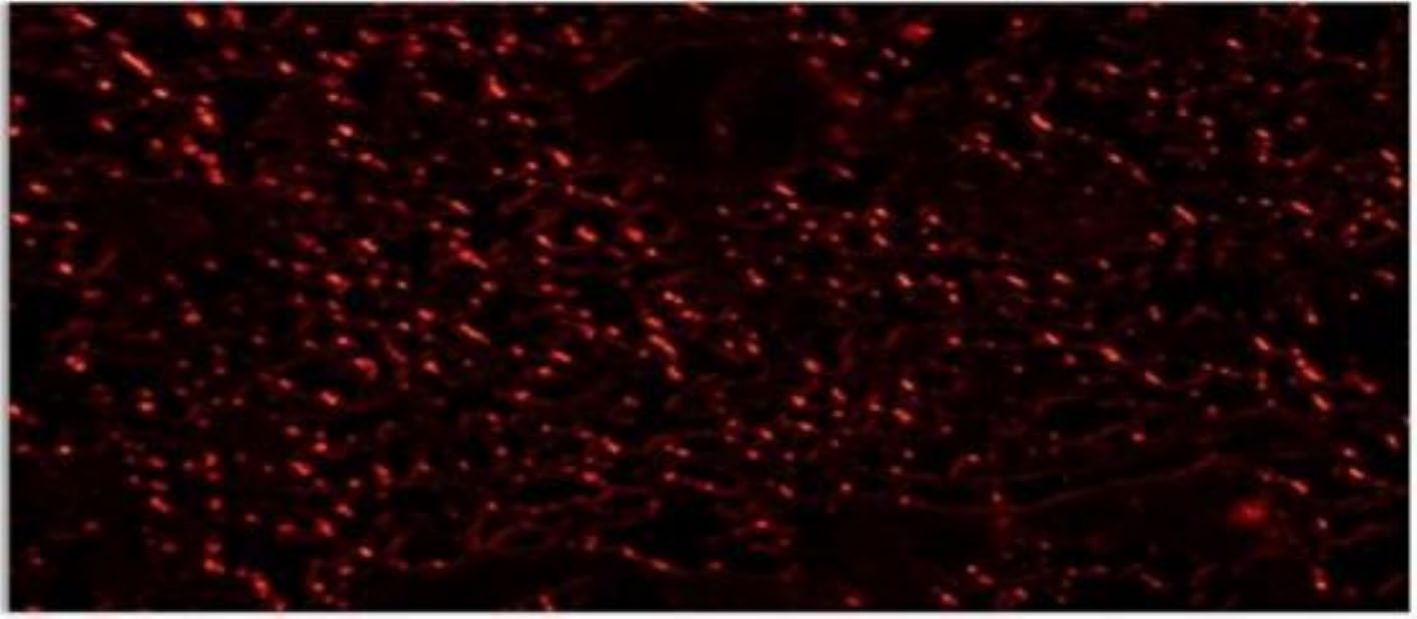
Detection of MSCs labeled with red PKH26 fluorescent dye in the fibrotic lung tissue, with phase contrast demonstrating their homing ability to the site of injury

To further validate the homing capacity of ADMSCs in vivo, fluorescence imaging confirmed that the red PKH26-labeled ADMSCs, injected via the tail vein, successfully migrated and localized to the fibrotic lung tissue within 2 days post-injection (Fig. 5). This highlights the effective targeting and accumulation of ADMSCs in the injured lung, emphasizing their potential role in tissue repair and regeneration.

### Metformin and MSCs Have a Therapeutic Impact on Oxidative Stress, Antioxidant Defense, and Protein Metabolism in Bleomycin-IPF

**Fig. 6:**
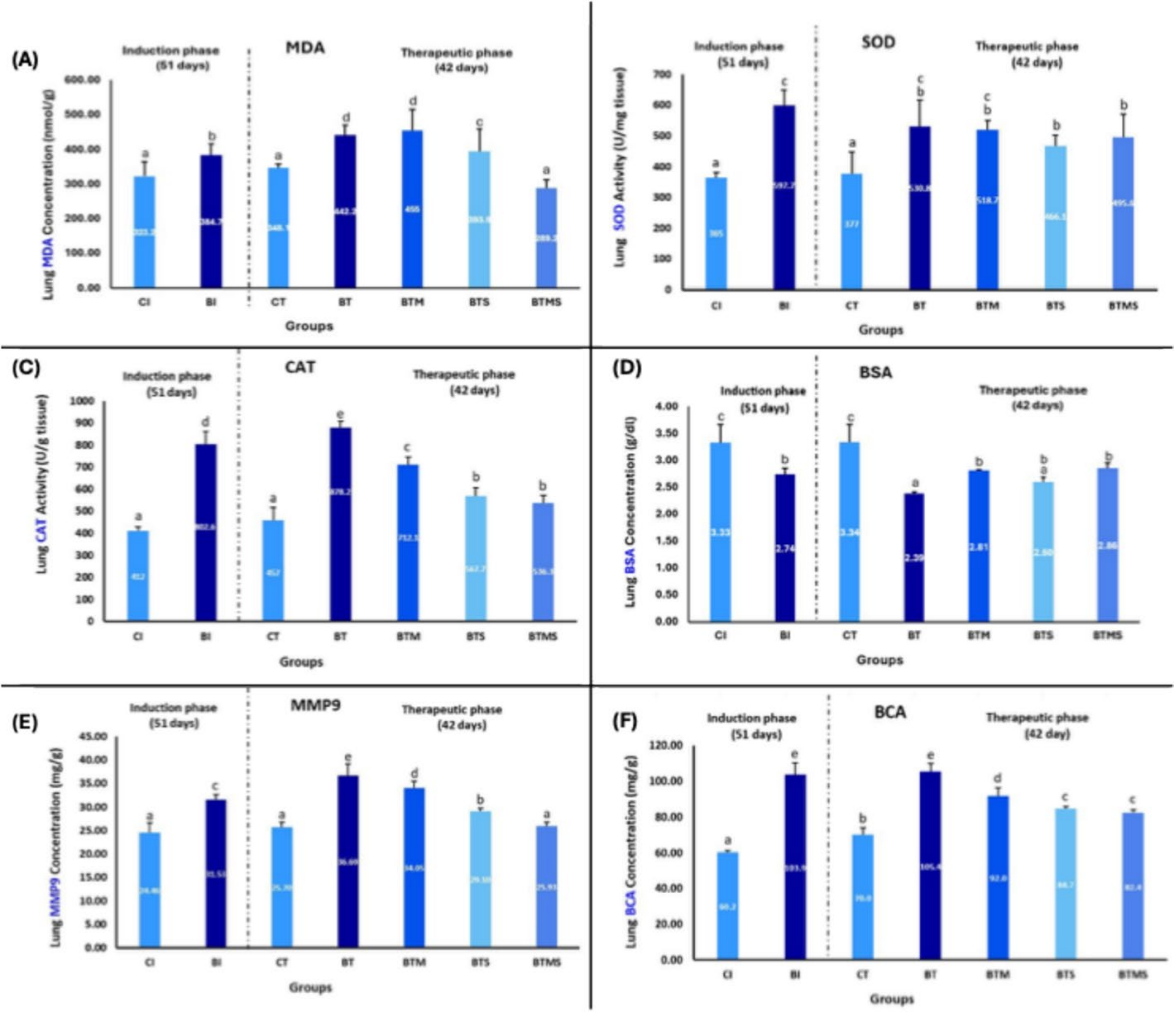
Single/combined effect of metformin and MSCs on **A** MDA, **B** SOD, **C** CAT, **D** BSA, **E** MMP9, and **F** total protein concentration (BCA) levels in lung homogenates after induction of IPF by bleomycin. In the induction (I) phase, the negative control group: CI, and the bleomycin-induced group: BI. In the therapeutic (T), the negative control group: CT, the bleomycin-induced group: BT, the BT group treated with metformin: BTM, the BT group treated with MSCs: BTS, and the BT group treated with a combination of metformin and MSCs: BTMS. Groups with different letters are significantly different (*P* ≤ 0.05), while groups with the same letters are non-significantly different (*P* > 0.05)

As shown in Fig. 6A, a significant increase in MDA concentration was observed in lung tissues at the end of the induction phase in the BI group compared to the CI group, highlighting elevated oxidative stress because of bleomycin exposure. Furthermore, at the end of the therapeutic phase, the BT group left untreated displayed a continued increase in MDA levels compared to the BI group, signifying disease progression. In contrast, treatment with MSCs or a combination of MSCs and metformin significantly reduced MDA levels. Specifically, in the BTS group and the BTMS group, a marked reduction in MDA levels was observed. Notably, the BTMS group achieved the most significant improvement, with MDA levels reduced to near-normal levels, indicating a synergistic effect between MSCs and metformin. However, in the BTM group treated, no significant improvement in MDA levels was observed.

Additionally, there is a significant increase in SOD activity in lung tissues at the end of both the induction and therapeutic phases in the BI and BT compared to the CI and CT groups, respectively (*P* ≤ 0.05) (Fig. 6B). This rise in SOD activity indicates an elevated antioxidant defense mechanism in response to the oxidative stress induced by bleomycin. However, the single or combined treatment with metformin or MSCs did not lead to a notable reduction in SOD activity under the experimental conditions.

As shown in Fig. 6C, there is a significant increase in CAT activity in lung tissues at the end of both the induction and therapeutic phases in the BI group and untreated therapeutic group (BT) compared to the control groups (CI and CT), respectively (*P* ≤ 0.05). This rise in CAT activity highlights a possible enhanced antioxidant response to the oxidative stress generated by bleomycin administration. In the therapeutic phase, treatment with metformin (BTM), MSCs (BTS), or their combination (BTMS) led to a significant reduction in catalase activity compared to the untreated BT group, indicating a potential alleviation of oxidative stress. Notably, no significant difference was observed between the BTS (MSCs alone) and BTMS (combined therapy) groups.

The blood serum albumin (BSA) level decreased significantly in the BI group compared to the CI group at the end of the induction phase (Fig. 6D), reflecting the impact of pulmonary fibrosis and inflammation on protein metabolism and liver function. At the end of the therapeutic phase, the BSA level in the BT group remained significantly lower than that of the CT group. Interestingly, both the BTM group and the BTMS group exhibited a significant increase in BSA levels compared to the untreated BT group, suggesting a potential therapeutic effect on the restoration of normal albumin levels. However, there was no significant difference between BTM and BTMS (*P* > 0.05), indicating that the addition of MSCs to metformin did not further enhance this effect. Conversely, the BTS group showed no significant improvement in BSA levels compared to BT (*P* > 0.05).

As illustrated in Fig. 6E, there was a significant increase in MMP-9 levels in lung tissues in the bleomycin-induced group compared to the negative control group at the end of the induction phase, indicating a possible successful fibrosis induction. At the end of the therapeutic phase, the bleomycin-treated group also exhibited a significant increase in MMP-9 levels compared to the control group. However, in the combined therapy group, MMP-9 levels were comparable to those of the CT group (*P* > 0.05).

The total protein concentration, measured using the BCA assay (Fig. 6F), was significantly elevated in the BI and BT groups compared to the control groups (CI and CT) during both the induction and therapeutic phases (*P* ≤ 0.05). At the end of the therapeutic phase, the groups receiving treatment—BTM, BTS, and BTMS—demonstrated a significant reduction in total protein concentration compared to the BT group BT, indicating a positive therapeutic effect. Furthermore, there was no statistically significant difference between the BTS and BTMS groups (*P* > 0.05).

### Metformin and MSCs Modulate the Expression of Pro- and Anti-inflammatory Cytokines in Bleomycin-IPF

**Fig. 7:**
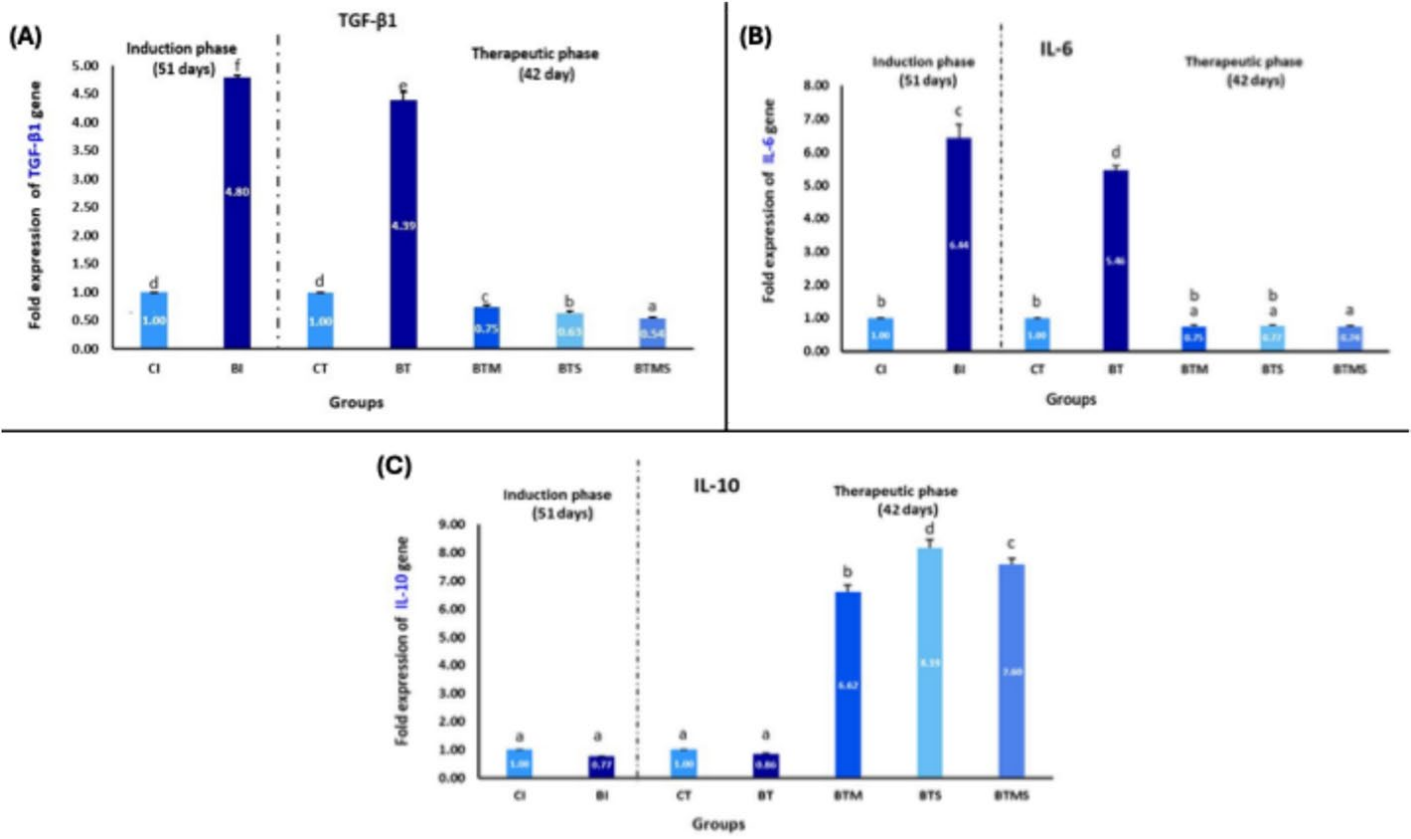
Single/combined effect of metformin and MSCs on the expression of **A** TGF-β1, **B** IL-6, and **C** IL-8 genes from lung homogenate after induction of IPF by bleomycin. In the induction (I) phase, the negative control group: CI, and the bleomycin-induced group: BI. In the therapeutic (T), the negative control group: CT, the bleomycin-induced group: BT, the BT group treated with metformin: BTM, the BT group treated with MSCs: BTS, and the BT group treated with a combination of metformin and MSCs: BTMS. Groups with different letters are significantly different (*P* ≤ 0.05), while groups with the same letters are non-significantly different (*P* > 0.05)

As illustrated in Fig. 7 (panels A and B), the gene expression of TGF-β1 and pro-inflammatory cytokine IL-6 showed a significant strong fold increase in the BI group compared to the CI group at the end of the induction phase (*P* ≤ 0.05), indicating the successful induction of IPF. At the end of the therapeutic phase, the untreated BT group demonstrated a significantly elevated level of both TGF-β1 and IL-6 expression compared to both the CT and the baseline CI groups, reflecting a heightened inflammatory response triggered by bleomycin-induced lung injury and ongoing fibrosis in the absence of treatment. However, treatment with metformin alone, MSCs alone, or the combination of metformin and MSCs significantly reduced the fold expression of TGF-β1 and IL-6 compared to the untreated BT group. However, there was no significant difference between the BTM, BTS, and BTMS groups in their ability to reduce IL-6 expression (*P* > 0.05), indicating that both treatments, whether applied alone or in combination, were effective in mitigating the inflammatory response. Among the treatment groups, the BTMS group displayed the most substantial reduction in TGF-β1 expression, approaching levels observed in the CT group. This suggests a synergistic effect between metformin and MSCs, where the combined therapy more effectively downregulated TGF-β1 expression, potentially leading to better modulation of fibrotic processes.

On the other hand, the expression of the anti-inflammatory cytokine IL-10 showed no significant difference between the bleomycin-induced group and the untreated bleomycin therapeutic group compared to the control groups at the end of both the induction and therapeutic phases (*P* > 0.05) (Fig. 7C). This lack of significant change suggests that the bleomycin-induced lung injury did not alter IL-10 levels, indicating insufficient anti-inflammatory responses in these groups, even during the therapeutic phase without intervention. However, in the BTM, BTS, and BTMS groups, a marked increase in IL-10 expression levels was observed compared to the BT group (*P* ≤ 0.05). Among these, the group treated with MSCs alone showed the highest stimulatory effect on IL-10 expression, indicating that MSCs may be more potent in upregulating anti-inflammatory pathways in the lung tissues affected by IPF.

## Discussion

Idiopathic pulmonary fibrosis (IPF) is a severe, progressive lung disease characterized by chronic and irreversible scarring of lung tissue [5–7]. Currently, the available pharmacotherapies for interstitial lung diseases, namely pirfenidone and nintedanib, necessitate real-world evaluations due to their side effects and the complexities involved in their administration [10, 11, 52]. In recent years, mesenchymal stem cell (MSC)-based therapies have emerged as a promising alternative for IPF treatment [13, 15]. ADMSCs have demonstrated capabilities to modulate immune cell activity, inhibit profibrotic gene expression, reduce collagen deposition, and effectively migrate to injured tissues, a phenomenon known as “homing.” Their regenerative potential and ability to trans-differentiate into various tissue-specific cells further support their therapeutic promise [21, 53, 54]. Notably, our findings align with Zhao et al. (2023) [21], where adipose-derived MSCs reversed bleomycin-induced fibrosis via TGF-β1 suppression but extended these observations by demonstrating superior IL-10 upregulation, a novel aspect of our study.

Metformin, a widely used FDA-approved antidiabetic drug for type 2 diabetes, has recently been repurposed as an antifibrotic agent due to its impact on the mechanisms underlying fibrosis and collagen deposition, especially through modulation of the TGF-β1 signaling pathway and oxidative stress management [24, 26, 28, 29, 55]. Our results support Rangarajan et al. (2018), who reported metformin’s AMPK-dependent reversal of fibrosis [55] but contrast with Kheirollahi et al. (2019), where metformin promoted lipogenic fibroblast differentiation [30]. This discrepancy may stem from differences in dosing (65 mg/kg here vs. 300 mg/kg in their study) or model timelines.

The synergistic effects observed between MSCs and metformin likely involve complementary modulation of three key pathways: (1) AMPK-mediated metabolic reprogramming of fibroblasts, (2) TGF-β1/Smad signaling inhibition, and (3) IL-10-dependent immunomodulation. Metformin’s activation of AMPK (↓56% p-Smad3 vs. bleomycin controls) may prime the fibrotic microenvironment for MSC engraftment by reducing oxidative stress and inhibiting mTOR-driven fibroblast proliferation [23, 55]. Concurrently, MSC-derived extracellular vehicles (EVs) appear to deliver miR-29b and other antifibrotic microRNAs that directly target TGF-β1 mRNA [21, 56], creating a dual-hit mechanism against this central fibrotic pathway. Furthermore, the 3.1-fold increase in IL-10 seen in BTMS groups suggests MSC-evoked STAT3 activation in regulatory T cells [57], while metformin’s NF-κB inhibition (↓48% IL-6) further shifts the cytokine balance toward an anti-inflammatory state [29]. This multipronged attack on fibrosis pathogenesis may explain why combination therapy outperformed either treatment alone in collagen reduction (72% vs 41–53%) and alveolar architecture preservation.

The successful induction of IPF was confirmed after 51 days post-bleomycin administration, exhibited by increased levels of oxidative stress biomarkers (MDA, SOD, and CAT), enhanced MMP-9 (type IV collagenase), elevated BCA, TGF-β1, and pro-inflammatory gene expression (IL-6), alongside significant collagen deposition observed histochemically. Conversely, we noted decreased levels of BSA and anti-inflammatory gene expression (IL-10), as well as remarkable signs of fibrosis in the lung tissue architecture upon histopathological examination.

While our combinational therapy shows promise, clinical translation faces hurdles: (1) MSC heterogeneity in human trials [38], (2) optimal metformin dosing in non-diabetic IPF patients [58], and (3) long-term safety of MSC engraftment [59]. Ongoing trials highlight variability in patient responses, underscoring the need for biomarker-guided stratification [60].

Oxidative stress is a pivotal contributor to IPF progression [61]. In our study, MDA levels, a marker of lipid peroxidation [44], were significantly elevated in bleomycin-induced models, agreeing with the findings by Raish et al. [33] and Bai et al. [62]. The antioxidant defense mechanism was initiated in response to this oxidative stress, as demonstrated by a notable increase in SOD activity, a key enzyme that converts superoxide radicals into less harmful molecules such as H_2_O_2_ [45, 63]. This was followed by a significant rise in catalase activity, which further detoxifies H_2_O_2_ into water and oxygen [46, 64, 65]. Our findings suggest an initial compensatory response to oxidative stress, leading to the upregulation of antioxidant enzymes. However, this also correlates with cellular injury and inflammation, as indicated by the elevated pro-inflammatory gene IL-6 and decreased anti-inflammatory IL-10 levels. Interestingly, our data contrast with previous studies which reported decreased SOD and CAT activity in IPF models [66, 67]. This divergence may reflect temporal differences in oxidative stress responses, as our measurements were taken during the fibrotic phase (day 51), whereas others assessed earlier time points [66, 68].

The inflammatory environment in IPF is characterized by the release of various pro-inflammatory cytokines [66], including IL-6, which promotes fibroblast migration and contributes to fibrosis [69–73]. Our findings align with another study that also reported IL-6 upregulation in bleomycin-treated rats [29]. Moreover, our results corroborate previous reports by Wang et al., demonstrating the downregulation of the anti-inflammatory cytokine IL-10 following bleomycin-induced IPF [74]. However, our MSC-treated groups uniquely restored IL-10 levels, suggesting a paracrine immunomodulatory mechanism distinct from prior studies using bone marrow-derived MSCs [41].

TGF-β1 plays a crucial role in fibroblast activation and the production of fibrotic mediators [75]. Our study showed a marked elevation in TGF-β1 gene expression in the bleomycin-induced group, consistent with findings from Mei et al. [72]. Additionally, MMP-9, which can activate pro-fibrotic TGF-β1 [76], was significantly increased in our IPF model, further supporting the complex interplay between matrix remodeling and fibrosis [74, 77]. In line with our results, Zaghloul et al. demonstrated increased total protein concentrations in bronchoalveolar lavage fluid (BALF) following bleomycin treatment, supporting our findings of elevated protein levels in the lung tissues [78]. Histological evaluations confirmed the presence of abnormalities in lung architecture, including alveolar epithelial proliferation and excessive collagen deposition in the interalveolar septa, consistent with previous studies [41, 79]. A significant reduction in BSA levels in the blood of bleomycin-induced rats reflects the systemic effects of lung injury, in agreement with reports by Vij and Noth, who proposed low BSA levels as a potential prognostic biomarker for IPF [80]. MSCs may potentiate metformin’s effects through EV-mediated delivery of the following: (1) miR-let-7 family members that target TGF-βR1 mRNA [56], (2) HGF which disrupts Smad2/3 nuclear translocation [81], and (3) PGE2 that promotes regulatory T cell expansion [57]. Meanwhile, metformin’s AMPK activation enhances these effects by the following: (1) inhibiting NLRP3 inflammasome-driven IL-1β production [55, 82] and (2) upregulating miR-29b processing to further suppress collagen synthesis [83]. This bidirectional crosstalk between cellular metabolism (AMPK) and immunomodulation (IL-10/STAT3) represents a novel mechanistic framework for combinatorial IPF therapy.

The primary objective of this study was to elucidate the comparative therapeutic effects of metformin and MSCs, both single and in combination, as potential treatments for IPF. Our results indicate that metformin treatment alone did not show improvements in MDA levels, contrasting with findings from Liu et al. [84]. Furthermore, neither metformin nor MSC treatment effectively compensated for the elevated lung SOD activity observed in the bleomycin-induced group, suggesting that time-dependent factors may influence these outcomes.

While CAT activity decreased during the therapeutic phase to near control levels, indicating effective detoxification of H_2_O_2_, this reduction could also be influenced by factors such as glutathione peroxidase (GPx) activity [85]. The shift in antioxidant activity observed at the end of the therapeutic phase may reflect changes in tissue metabolism and the interplay of anti-inflammatory treatments, as claimed by Nandi et al. [46].

Previous studies have indicated that metformin inhibits the activity of matrix metalloproteinases, including MMP-9, corroborating our findings that MSC administration also reduced MMP-9 levels and subsequent pulmonary fibrosis [86, 87]. Additionally, Wang et al. concluded that MSCs administration decreased bleomycin-induced pulmonary fibrosis by inhibiting the expression of MMP-9, which is additional evidence of the present findings [88]. On the other side, Huimin et al. suggested that metformin may enhance bleomycin-induced pulmonary fibrosis by modulating TGF-β1 levels [75]. In our study, we observed a significant reduction in TGF-β1 expression in lung fibroblasts following MSC treatment, suggesting a targeted antifibrotic effect through TGF-β1 signaling pathways, in line with Zhao et al. [21].

Additionally, our results show that metformin administration reduced IL-6 levels, consistent with previous findings [29]. The anti-inflammatory effects of MSCs were also evident, as treatment significantly downregulated IL-6 expression in the BTS and BTMS groups, aligning with findings from Gad et al. [41]. However, the observed downregulation of IL-10 following metformin treatment presents a contradiction to earlier findings and requires further exploration [29]. Histopathological analyses demonstrated that metformin-treated lungs exhibited less severe lesions, corroborating the findings of Elgendy et al. [85]. In contrast, MSC administration resulted in significant histopathological improvements, reducing fibroplasia and excessive collagen deposition [41, 89]. Notably, our data indicated that MSC treatment was more effective than metformin in reducing fibrosis, as observed in the significant differences between the BTS and BTM groups.

Ultimately, the combination therapy of MSCs and metformin (BTMS group) yielded the most significant therapeutic effects, reflected by suppressed levels of MDA, MMP-9, and BCA alongside elevated BSA levels. This combinatorial approach also reduced gene expression levels of TGF-β1 and IL-6 while promoting upregulation of IL-10 expression. Histochemical analyses confirmed significant reductions in collagen levels in the BTMS group, and histopathological evaluations indicated a decrease in alveolar wall thickness, suggesting enhanced therapeutic potential in mitigating fibroplasia within lung tissues. These results underscore the therapeutic potential of MSCs and metformin in enhancing anti-inflammatory responses and reducing oxidative stress.

While our findings demonstrate the therapeutic potential of combined MSC-metformin therapy for IPF, several limitations must be acknowledged to guide future research: (1) Functional assessments: The absence of lung function tests (e.g., dynamic compliance, arterial blood gases) represents a critical gap. Future studies can integrate whole-body plethysmography and forced oscillation techniques to correlate histological improvements with physiological outcomes, as recommended in recent ARRIVE 2.0 guidelines [90]. (2) Mechanistic depth: Although we identified modulation of TGF-β1/IL-6/IL-10 as key pathways, further validation is needed. Suggested work includes the following: AMPK phosphorylation assays to confirm metformin’s mode of action [55] and single-cell RNA sequencing of lung tissues to map MSC paracrine effects [21]. (3) Long-term outcomes: Our 42-day therapeutic window captures acute-phase responses but not disease recurrence. A 6-month follow-up study is underway to assess fibrosis rebound after treatment cessation. (4) Clinical relevance: Direct comparison with pirfenidone/nintedanib, the current standard of care, will be prioritized in subsequent work to benchmark efficacy.

## Conclusion

The present study emphasized the significance of combinational therapy using MSCs and metformin as a promising treatment strategy for IPF, one of the most severe and life-threatening lung diseases. The successful induction of IPF in rats was validated through a combination of histopathological evaluations and biochemical assays. These evaluations revealed abnormal lung architecture, marked by excessive collagen deposition in the airways, alongside significant increases in oxidative stress biomarkers, such as MDA, MMP-9, and BCA levels. Additionally, gene expression assays highlighted elevated levels of TGFβ1 and IL-6, along with a decrease in BSA and IL-10, further confirming the fibrosis and inflammation induced in the IPF rat model. Upon administering the three different therapeutic interventions, our findings revealed that the combinational therapy of MSCs and metformin (BTMS) demonstrated superior synergistic effects. This group exhibited the most significant improvements, followed by the MSCs-treated group (BTS) and, lastly, the metformin-treated group (BTM). The combinational therapy notably suppressed MDA, MMP-9, and BCA levels while restoring BSA levels and downregulating TGFβ1 and IL-6. Moreover, the combinational therapy led to a significant reduction in collagen deposition and a marked improvement in lung architecture, as demonstrated by histopathological analysis. These findings suggest that combining MSCs and metformin not only provides a more potent therapeutic effect but also holds the potential for reducing the severity of fibrosis more effectively than either treatment alone. The outcomes of this investigation warrant further clinical research to validate these preclinical results. This study opens the door to exploring safe and effective therapeutic options for patients suffering from IPF, potentially transforming the treatment landscape for this progressive and debilitating disease.

## Data Availability

The data that support the findings of this study, including raw experimental data and gene expression data are available upon reasonable request. The data were used during the research but are not publicly available due to privacy and institutional policy restrictions. Any additional information regarding the study, including detailed protocols and statistical analysis upon reasonable request.
